# Indications of Arthroscopy in the Treatment of Patellar Instability in Children

**DOI:** 10.7759/cureus.58437

**Published:** 2024-04-17

**Authors:** Nam Q Vo, Trung H Nguyen, Thai H Phan

**Affiliations:** 1 Pediatric Orthopaedic Department, Hospital for Traumatology and Orthopaedics, Ho Chi Minh, VNM

**Keywords:** medial reefing, lateral release, trochlear dysplasia, patellofemoral instability, medial patellofemoral ligament

## Abstract

Purpose

Understanding the relevant risk factors for patellar instability and the clinical and radiographic tests necessary to determine optimal treatment. This case series intends to evaluate arthroscopic indications in the treatment of patellar instability in children.

Methods

From 2013 to 2021, 33 patients (seven to 16 years of age) with 35 knees sustaining first-time dislocation with loose bodies, recurrent dislocation or subluxation, and habitual dislocation were arthroscopically operated on according to the flow chart. Periods of follow-up were two to 10 years (avg. 5.5 years). Follow-up assessment included the recurrence, complications (joint stiffness and excessive reduction), and the final function outcomes by using the Kujala score.

Results

Among 35 knees, there were two (5.7%) first dislocations, 30 (85.7%) recurrent dislocations, and three (8.6%) habitual dislocations, lateral release 27/35 (77.1%), medial reefing 23/35 (65.7%), reconstruction of the medial patellofemoral ligament (MPFL) 12/35 (34.3%). The major complication was a knee of extensive stiffness after medial reefing and lateral release. Recurrence was in 4/35 (11.4%) of knees, not correlated to lateral release (p=0.21), medial reefing, or reconstruction of MPFL (p=0.07); in about 23 knees of medial reefing, recurrence was significantly correlated to number of knots (p=0.045). The final functional results according to Kujala were 88-100 (avg. 95.5).

Conclusions

This study showed the role of arthroscopy in both medial reefing and reconstruction of MPFL in children by low recurrence rate and high Kujala score at final follow-up. There was no significant correlation between recurrence and the procedures as arthroscopic indications counted on the flow chart.

## Introduction

With a frequency of 43/100000 in children, patellofemoral instability (PFI) is quite common [1]. The recent research conducted by Abbassi et al. revealed that 31% of adolescents with knee injuries were diagnosed with PFI [2]. In children aged 10 to 14 years, PFI is the most common cause of knee joint effusion, whereas anterior cruciate ligament tears are more prevalent in adolescents aged 15 to 18 years [2]. PFI can either be an isolated PFI (an acute dislocation or an initial dislocation) or a chronic dislocation. Two recent studies in children with initial acute patellar dislocation have shown a recurrence rate of 30-38%; the recurrence rate is higher in the 11-13 age group with open growth plates and dysplasia [1,3]. Patellar dislocations, and symptomatic osteochondral lesions, reduce activity levels and impair the quality of life in patients with recurrent PFI; moreover, chondral defects and recurrent damage to the articular surface might increase the risk of early-onset osteoarthritis.

Various surgical approaches for treating recurrent patellar dislocation are described. Indication of surgical procedures depends on each patient and the pathoanatomy causing recurrent PFI [4,5]. In the majority of cases, reconstructing the medial patellofemoral ligament (MPFL) is preferred over medial reefing because the medial soft tissue system is chronically damaged and usually insufficient to prevent lateral dislocation. The hamstring tendon, patellar tendon, adductor magnus, quadriceps tendon, and allografts are all viable graft options for reconstruction of the MPFL. D'Ambrosi et al., 2021, reviewed the literature on complications and recurrences following MPFL reconstruction in children, revealing a recurrence rate of 18 out of 352 knee joints (5.1%); there were no serious complications, but high-quality evidence was essential to better assess the long-term results [6]. A single MPFL reconstruction may not be enough to prevent recurrence if other risk factors such as patella alta, patellar tilt, an increased Q angle, a tibial tuberosity-trochlear groove (TT-TG) distance greater than 20 mm, or trochlear dysplasia are present [7]. On the other hand, some studies reported acceptable results in patients with recurrent PFI managed by arthroscopic lateral release and medial reefing [8-10].

Despite arthroscopy being not strongly mentioned lately, it has a role in assessing associated injuries, performing lateral release, and medial reefing, as well as positioning during MPFL reconstruction. Consequently, the objective of this article is to evaluate the indications for arthroscopy in the treatment of PFI in children.

## Materials and methods

Subjects

Between 2013 and 2021, 160 patients with PFI were operated on. Among these, 40 patients had arthroscopy for one of the following indications: first-time dislocation with loose bodies, recurrent dislocation or subluxation, and habitual dislocation. This case series study included patients who had no prior surgery, a minimum follow-up of two years, and no anomalies in the femur and tibial alignment; patients were excluded if MRI was not available, and evaluation indices were inadequate. Finally, 33 patients (35 knee joints) aged from seven to 16 years of age were cross-sectionally recruited by convenience sampling with a follow-up period of two to 10 years (avg. 5.5 years).

The study was conducted in accordance with the Declaration of Helsinki and approved by the Ethics Committee of the Hospital for Traumatology and Orthopaedics with decision number 794/QD-BVCTCH signed on September 21st, 2020 after the project of this study had been officially presented. Informed consent has been obtained and signed by the parents of all patients to publish this paper.

Methods

The clinical variables include the joint laxity, the J-sign, the apprehension test, lateral tightness, and the lateral patellar glide (2/4, 3/4). The MRI variables include location of MPFL injury (none, patellar, femoral, and combined), trochlear groove dysplasia (normal, mild >145 degrees, and severe ≥180 degrees), patellar position (normal, tilted, and subluxated), C-D index (Caton-Deschamps index) (≤1.2, >1.2), and TT-TG distance (≤ 1.2 cm, >1.2-<2.0 cm, ≥2.0 cm).

**Figure 1:**
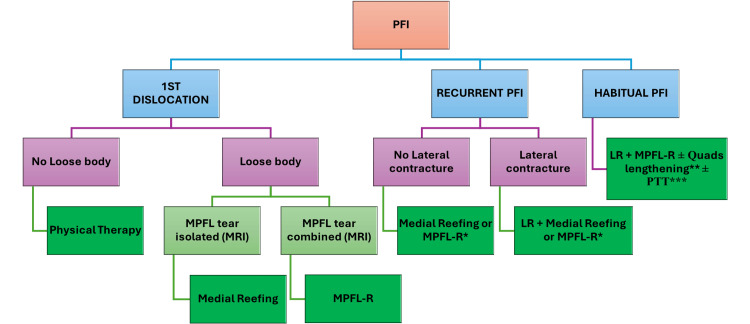
Indications of arthroscopy The figure is produced by the authors and not borrowed from any external source. *MPFL-R if matured or high-grade trochlear dysplasia **Quads lengthening if C-D >1.2 or limited flexion after MPFL-R ***PTT if TT-TG ≥20 mm or reconstruction not sufficient PFI, patellofemoral instability; LR, lateral release; MPFL, medial patellofemoral ligament

Arthroscopic indications are followed by a flowchart (Figure 1), including medial reefing, MPFL reconstruction, and lateral release. Medial reefing comprises three to four knots using PDS 0 or Hifi 2.0. MPFL reconstruction uses the quadriceps tendon (if under 10 years old) or semitendinosus tendon via a patellar tunnel (if over 10 years old); MPFL reconstruction is indicated if the patient is matured or has high-grade trochlear dysplasia. Lengthening of the quadriceps tendon, transfer of the patellar tendon, or both are performed when indicated.

Follow-up assessment included the recurrence, complications (joint stiffness and excessive reduction), and the final function outcomes by using the Kujala score [11]. Criteria of the postoperative recurrence were recurrent PFI, habitual PFI, or permanent PFI.

Statistical analysis

The duration of follow-up was calculated from the date of surgical treatment. Data was collected and analyzed using STATA software version 19 (StataCorp LLC; College Station, TX). X^2^ tests and Fisher exact tests were used for significance; a p-value <0.05 was considered significant.

## Results

In this study, girls were dominant with 27/35 (74.2%) and boys 8/35 (25.8%). Clinical signs were shown in Table 1 with joint laxity in 8/35 cases (22.9%), apprehension signs in 14/35 cases (40.0%), lateral contracture that was frequent in 22/35 cases (62.9%), and lateral patellar glide 2/4 in 16/35 cases (45.7%) and 3/4 in 19/35 cases (54.3%).

**Table 1:** Clinical signs Lateral contracture was frequent in 22/35 cases (62.9%).

	Cases (%)
Joint laxity	8/35 (22.9%)
Apprehension sign	14/35 (40.0%)
Lateral contracture	22/35 (62.9%)
Lateral patellar glide	
2/4	16/35 (45.7%)
3/4	19/35 (54.3%)

Abnormalities on MRI are presented in Table 2. Location of MPFL injury was patellar of 7/35 cases (20.0%), femoral of 4/35 cases (11.4%), and combined of 6/35 (17.2%) cases, but not defined in most cases with 18/35 (51.4%). Trochlear dysplasia was constant as mild in 23/35 cases (65.7%) and severe in 12/35 cases (34.3%). Additionally, position of patella was normal in 8/35 cases (22.9%), tilting in 8/35 cases (22.9%), and subluxated in 19/35 cases (54.2%); C-D index was ≤1.2 in 25/35 cases (71.4%) and >1.2 in 10/35 cases (28.6%); TT-TG distance was ≤1.2 cm in 23/35 cases (65.7%) and >1.2-<2.0 cm in 10/35 cases (28.6%), and ≥2.0 cm in 2/35 cases (5.7%).

**Table 2:** Abnormalities on MRI The location of MPFL injury was not defined in most cases with 18/35 (51.4%). MPFL, medial patellofemoral ligament; C-D, Caton-Deschamps; TT-TG, tibial tuberosity-trochlear groove

	Cases (%)
Location of MPFL injury	
None	18/35 (51.4%)
Patellar	7/35 (20.0%)
Femoral	4/35 (11.4%)
Combined	6/35 (17.2%)
Trochlear dysplasia	
Mild	23/35 (65.7%)
Severe	12/35 (34.3%)
Position of patella	
Normal	8/35 (22.9%)
Tilting	8/35 (22.9%)
Subluxated	19/35 (54.2%)
C-D index	
≤1.2	25/35 (71.4%)
>1.2	10/35 (28.6%)
TT-TG distance (cm)	
≤1.2	23/35 (65.7%)
>1.2-<2.0	10/35 (28.6%)
≥2.0	2/35 (5.7%)

Among these 35 knees, acute dislocations occurred in two cases (5.7%), habitual PFI in three cases (8.6%), and recurrent PFI was most frequent in 30 cases (85.7%). In arthroscopically surgical techniques, the lateral release was in 27/35 cases (77.1%), medial reefing in 23/35 cases (65.7%), and MPFL reconstruction in 12/35 cases (34.3%). Besides, lengthening of the quadriceps tendon was only in one case of habitual PFI.

When these patients were followed up, we found that one patient sustained knee stiffness after lateral release and medial reefing, which was managed by arthrotomy and lengthening of the quadriceps tendon. Notably, recurrence was observed in four of 35 cases (11.4%) of knees, not correlated with lateral release (p=0.21) and medial reefing or MPFL reconstruction (p=0.07) (Table 3). Of the 23 knees treated by medial reefing, recurrence occurred in 2/3 cases with two knots, 1/15 cases with three knots, and 0/5 cases with four knots; the difference was significant (p=0.045) (Table 4). As a final follow-up, the clinical outcome was evaluated by the Kujala score of 88-100 (avg. 95.5).

**Table 3:** The correlation between procedures and recurrence Recurrence was not correlated with medial reefing or MPFL reconstruction (p=0.07). MPFL: medial patellofemoral ligament

	Medial reefing	MPFL reconstruction	p-value
Recurrence	3/23 (13.0%)	1/12 (8.3%)	0.07

**Table 4:** Correlation between the number of knots and recurrence in the medial reefing group By medial reefing, recurrence was significantly correlated with number of knots (p=0.045).

	2 knots	3 knots	4 knots	p-value
Recurrence	2/3 (66.7%)	1/15 (11.1%)	0/5 (0%)	0.045

## Discussion

In this study, we aimed to evaluate the role of arthroscopy in the treatment of PFI in children. Specifically, our objectives included assessing surgical indications, procedures performed (such as lateral release, medial reefing, and MPFL reconstruction), recurrence rates, complications, and functional outcomes following the arthroscopic intervention.

For arthroscopic indications, many clinical and radiological signs have to be examined. Most of these cases were recurrent PFI with 30/35 cases (85.7%); so, it was essential to evaluate lateral retinacular tightness for the indication of arthroscopic lateral release. In addition to examining the lateral tightness by tilt or subluxation of the patella on MRI in 27/35 cases (77.1%), clinical signs of lateral tightness were also essential in 22/35 cases (62.9%). Besides maturity, the level of trochlear dysplasia on MRI affected the choice between lateral reefing and MPFL reconstruction according to the flowchart (Figure 1); actually, severe dysplasia accounted for 12/35 (34.3%) cases, whereas MPFL reconstruction is generally equivalent in 12/35 (34.3%) cases. Lippacher et al. in 2012 suggested that lateral X-ray does not define the severity of dysplasia compared to axial MRI [12].

We are also using MRI to assess the location of injury of MPFL, but there are up to 18/35 (51.4%) cases without clearance of the location of the injury, primarily because 94.3% of cases involved recurrent PFI or habitual PFI. In 2018, VQD Nam et al. conducted a literature review on MPFL injuries seen on MRI, revealing that the location and kind of MPFL injuries are not consistently identified on MRI, and numerous studies have found that injuries often occur at the patellar attachment site in children; moreover, the studies using MRIs to examine MPFL injuries in recurrent PFI are currently lacking [13].

By comparing MPFL reconstruction and medial reefing, Table 3 illustrated the recurrence rates of the two groups, which were not substantially different at 8.3% and 13.0%, and quite low. Song et al. compared arthroscopic medial reefing and MPFL reconstruction without bone abnormalities by reviewing 13 papers comprising 417 patients with at least a two-year follow-up; the results were encouraging with a recurrence rate of <10% regardless of method, graft type, or time of follow-up [14]. In 2021, Herdea et al. showed that MPFL reconstruction increased the quality of life among the pediatric population more than lateral release and medial reefing; however, their procedures were open [15].

Although there have been fewer studies on arthroscopic medial retinacular reefing, the outcomes have been positive, especially with long-term follow-up. Ali et al. performed lateral release and medial reefing on 36 knees of 35 patients, with an average follow-up of 51 months; the authors reported fewer complications and faster recovery [8]. In 2021, Nha et al. published the results of arthroscopic lateral release and medial reefing in 25 patients of recurrent PFI aged 18.3±4.8 years, with a follow-up period of 3.8-12.2 years; there was just one case of recurrence and two cases showed signs of patellofemoral degeneration [10]. On the other hand, Schorn et al. reported the results of medial reefing and lateral release in 43 knees of 38 patients aged nine to 44 years, during a follow-up period of 4.7-14.7 years [16]. Complications were reported in 34 knees (79%), with a 16% recurrence rate after one year and 52% after five years; the authors concluded that this method might not be enough for treating chronic instability with long-term follow-up. Their research had a higher recurrence rate than ours (11.4%) despite our follow-up duration being shorter (two to 10 years). Additionally, Table 4 showed a significant association (p=0.045) between the number of knots and the recurrence rate. We found that it is preferable to use four knots and that using two is not advised. Xu showed that there was no recurrence in 15 patients who had three to four knots despite following up for only one to two years [9].

The recurrent case in the MPFL reconstruction group was with habitual PFI, which might require lengthening of the quadriceps tendon and transfer of the patellar tendon according to our arthroscopic indication flowchart; both procedures were carried out for this patient later.

The Kujala score is frequently used nowadays for evaluating knee joint function. Although it was not initially analyzed, the final follow-up results based on this score are extremely high. Five of the cases with a score below 90 were in the group of MPFL reconstruction with the semitendinosus tendon, even though the scar was smaller than in a group of MPFL reconstruction with the quadriceps tendon.

However, this study has limitations including a lack of assessment of limb alignment on EOS, restricted data, and no consensus among surgeons on the evaluation of indexes and indications.

## Conclusions

This study showed the role of arthroscopy in both medial reefing and reconstruction of MPFL in children by low recurrence rate and high Kujala score at final follow-up. There was no significant correlation between recurrence and the procedures as arthroscopic indications counted on the flowchart. The flowchart has to be consented to and refined by further studies.
